# Supplementary material for: Simple Carbohydrate Derivatives Diminish the Formation of Biofilm of the Pathogenic Yeast Candida albicans
Source: Antibiotics (Basel). 2019 Dec 30;9(1):10. doi: 10.3390/antibiotics9010010 (PMC7167926; doi:10.3390/antibiotics9010010)
Supplement: Supplementary file 1 [file antibiotics-09-00010-s001.zip › SupportingInformation/RevSuppInf.docx]

Supporting Information

Simple Carbohydrate Derivatives Diminish the Formation of Biofilm of *Candida albicans*

Olena P. Ishchuk^a, b^, Olov Sterner^b^, Ulf Ellervik^b^, Sophie Manner^b*^

*^a^* Department of Biology, Lund University, Sölvegatan 35, SE-223 62 Lund, Sweden.

*^b^* Centre for Analysis and Synthesis, Centre for Chemistry and Chemical Engineering, Lund University, P.O. Box 124, SE-221 00 Lund, Sweden.

* Corresponding author. Centre for Analysis and Synthesis, Centre for Chemistry and Chemical Engineering, Lund University, P.O. Box 124, SE-221 00 Lund, Sweden. *E-mail address:* sophie.manner@chem.lu.se.

[1. 1-NMR, 13C-NMR 2](#_Toc529911207)

[Benzyl (2,3,4-tri-O-acetyl) α-L-fucopyranoside 2](#_Toc529911208)

[Benzyl (2,3,4-tri-O-acetyl) β-L-fucopyranoside 3](#_Toc529911209)

# 1. ^1^-NMR, ^13^C-NMR

## Benzyl (2,3,4-*tri*-*O*-acetyl) α-L-fucopyranoside

## Benzyl (2,3,4-*tri*-*O*-acetyl) β-L-fucopyranoside
