# Supplementary figures and images for: Simple Carbohydrate Derivatives Diminish the Formation of Biofilm of the Pathogenic Yeast Candida albicans
Source: Antibiotics (Basel). 2019 Dec 30;9(1):10. doi: 10.3390/antibiotics9010010 (PMC7167926; doi:10.3390/antibiotics9010010)

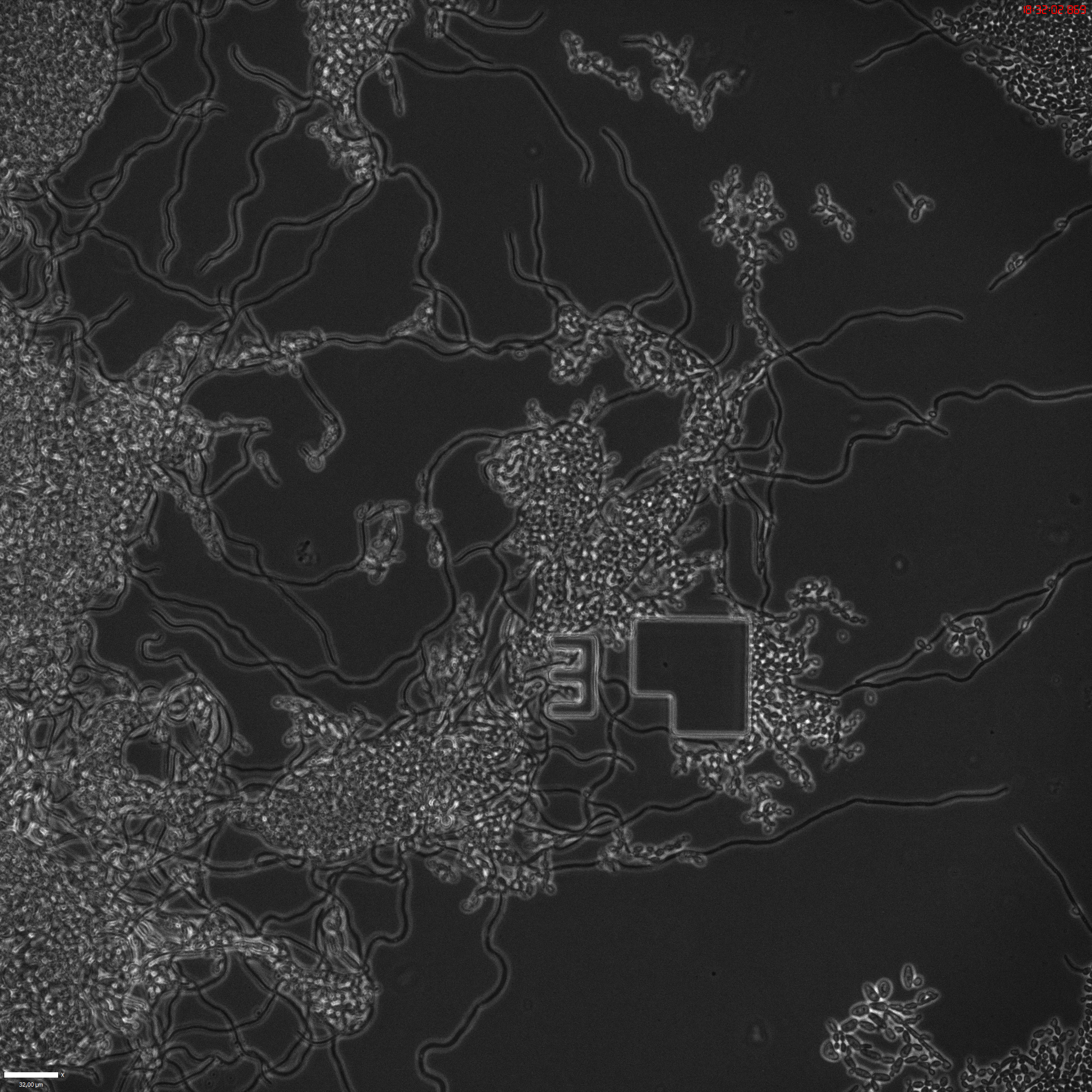

Supplement: Supplementary file 1 [file antibiotics-09-00010-s001.zip › SupportingInformation/Figure3bHighRes18h.tif]

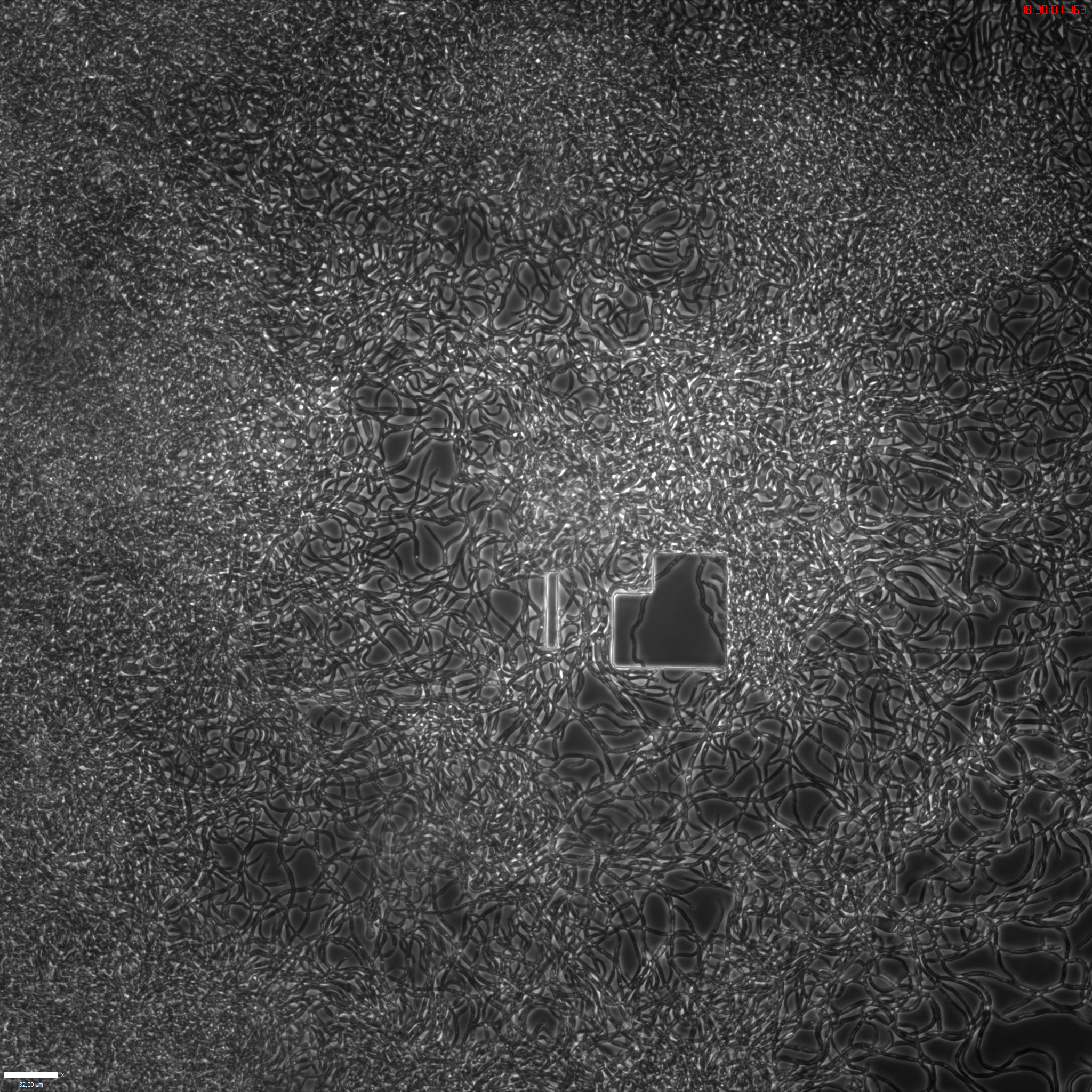

Supplement: Supplementary file 1 [file antibiotics-09-00010-s001.zip › SupportingInformation/Figure3aHighRes18h.tif]

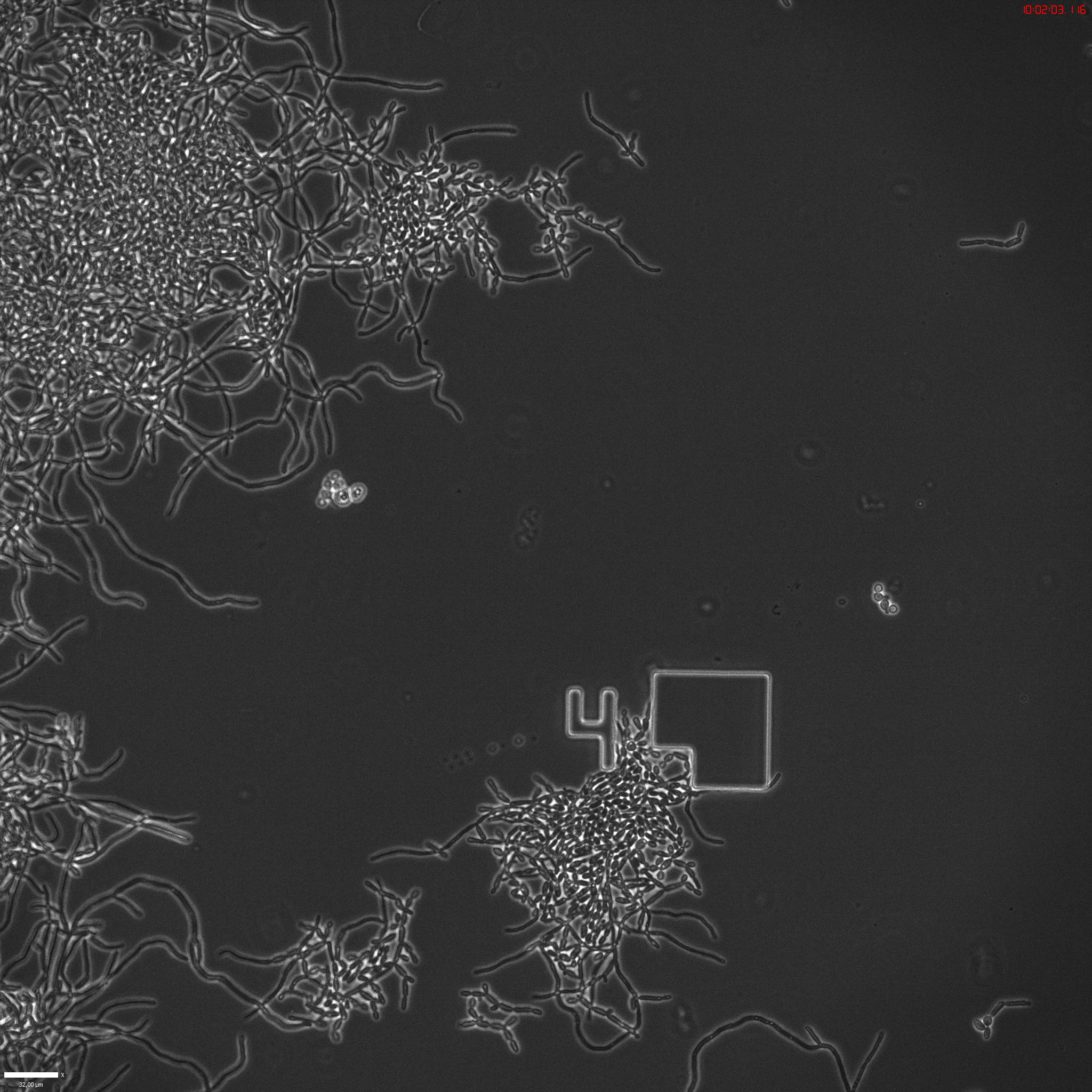

Supplement: Supplementary file 1 [file antibiotics-09-00010-s001.zip › SupportingInformation/Figure3cHighRes10h.tif]

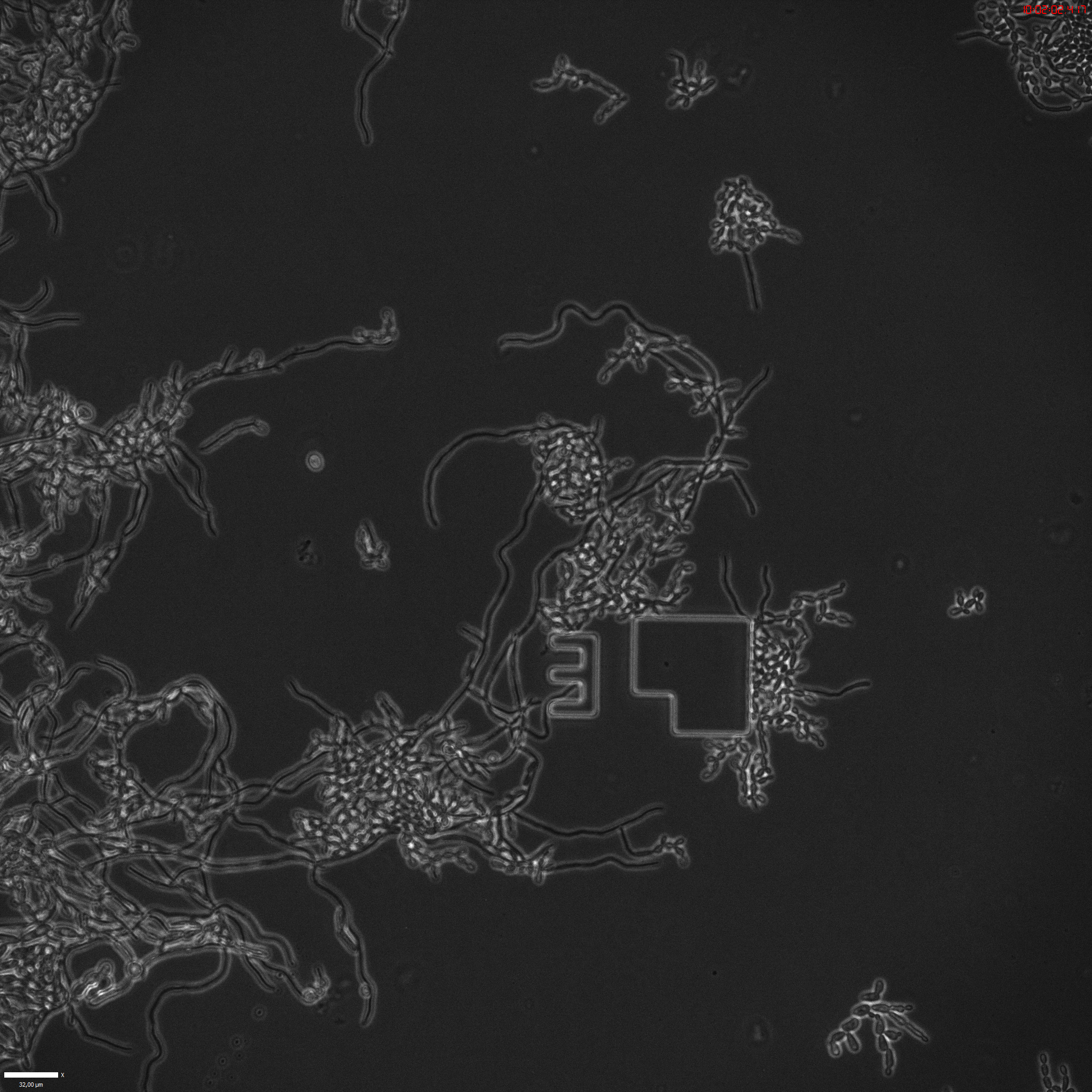

Supplement: Supplementary file 1 [file antibiotics-09-00010-s001.zip › SupportingInformation/Figure3bHighRes10h.tif]

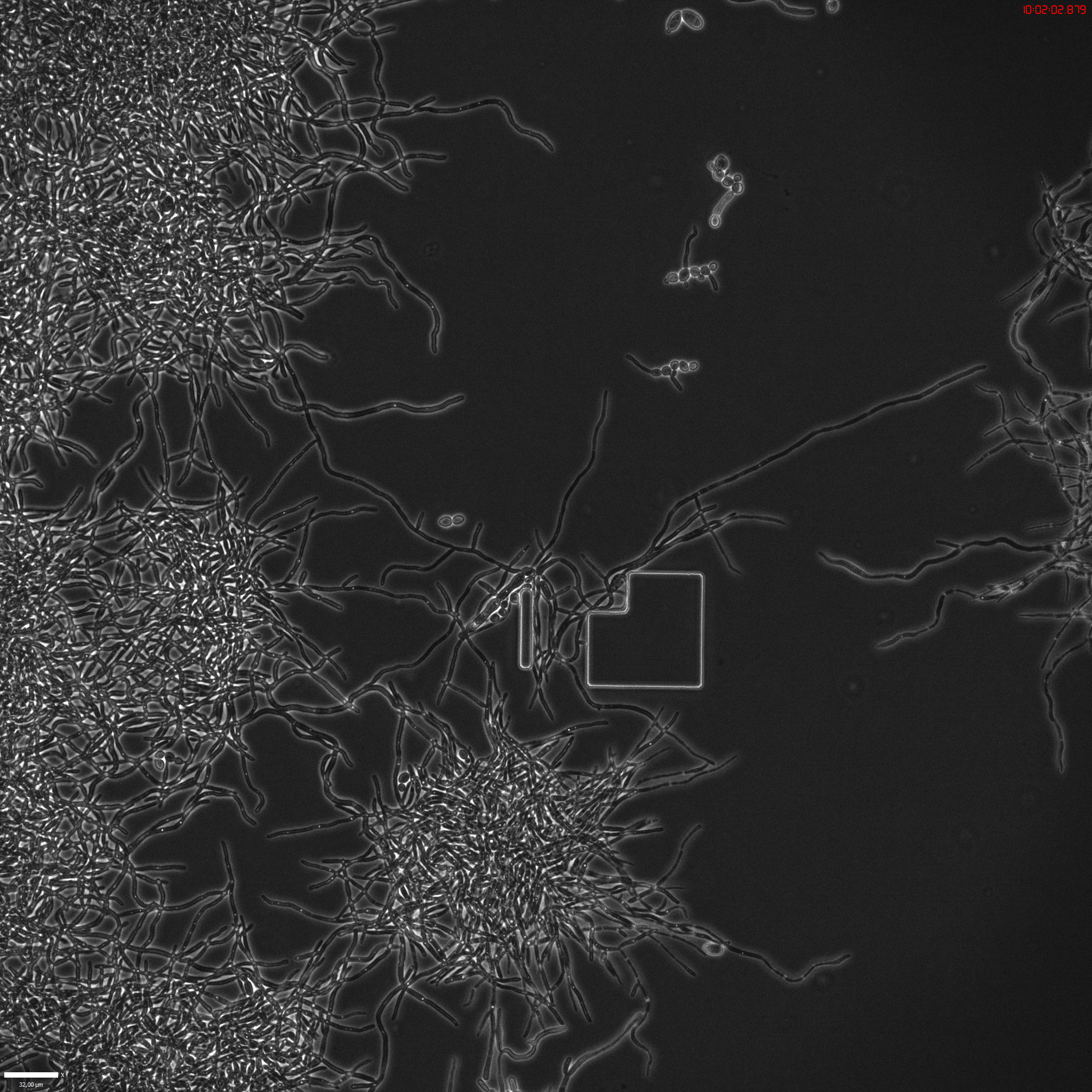

Supplement: Supplementary file 1 [file antibiotics-09-00010-s001.zip › SupportingInformation/Figure3aHighRes10h.tif]

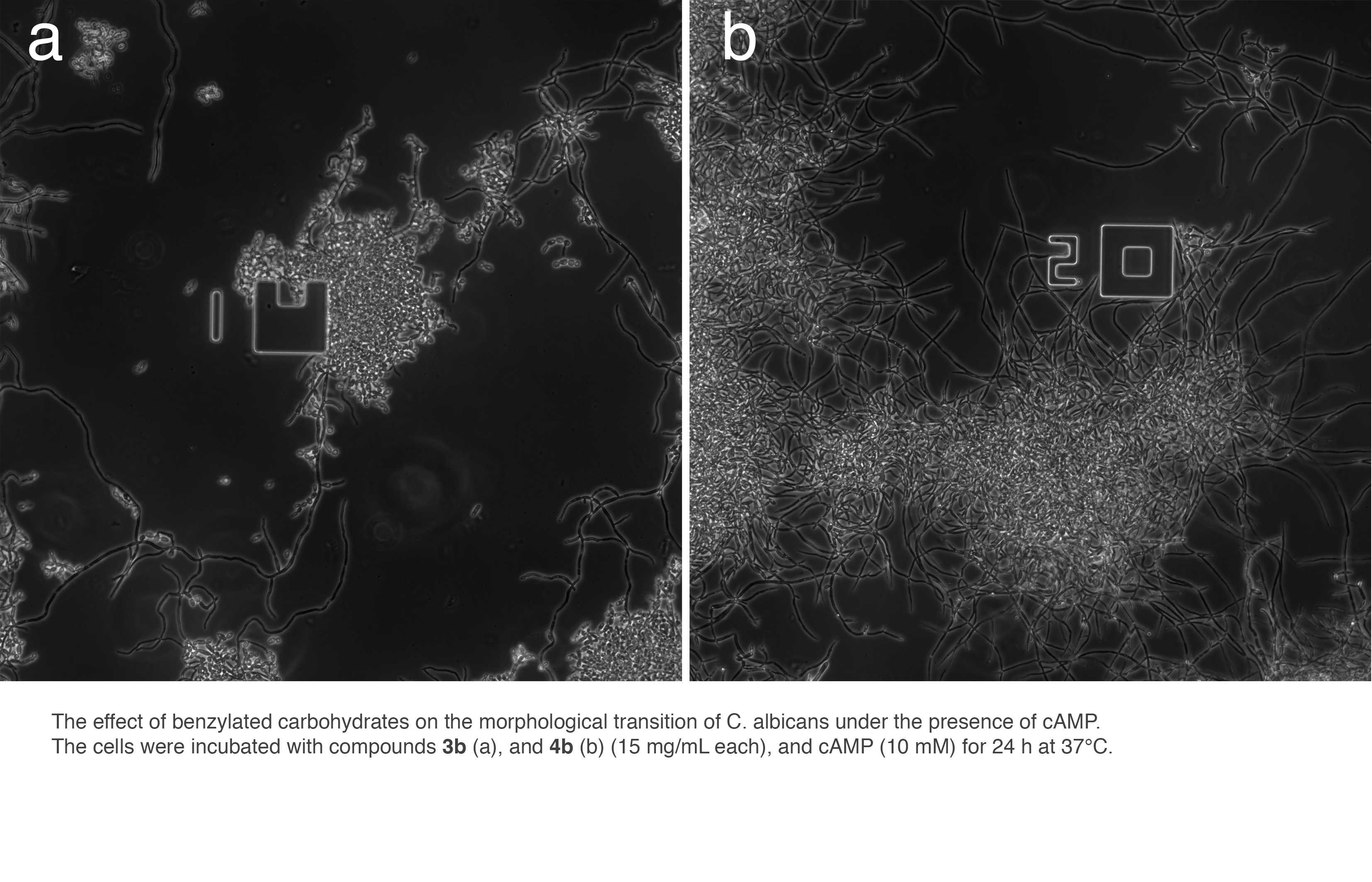

Supplement: Supplementary file 1 [file antibiotics-09-00010-s001.zip › SupportingInformation/cAMP3band4b.jpg]

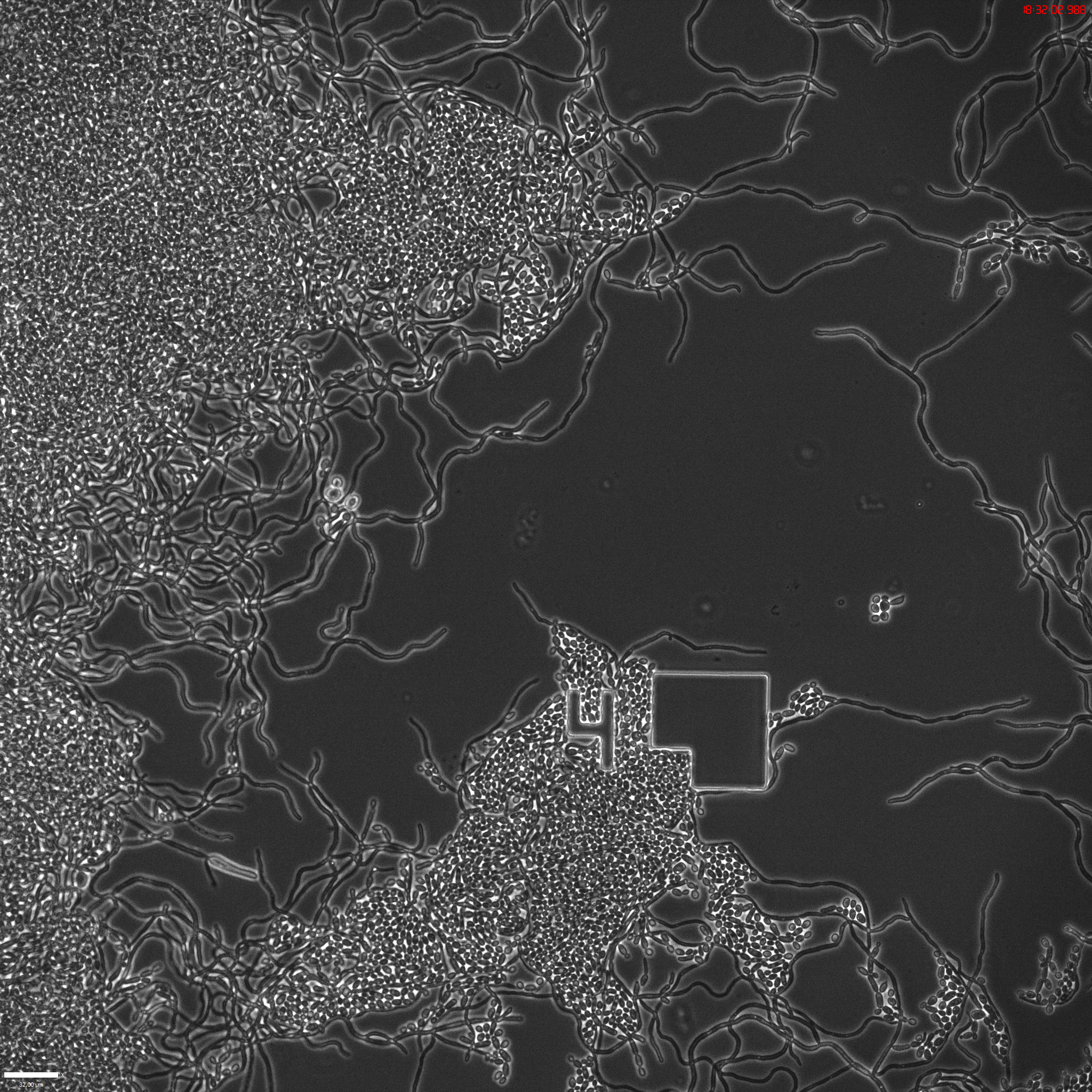

Supplement: Supplementary file 1 [file antibiotics-09-00010-s001.zip › SupportingInformation/Figure3cHighRes18h.tif]
